# The Improvement of Laparoscopic Surgical Skills Obtained by Gynecologists after Ten Years of Clinical Training Can Reduce Peritoneal Adhesion Formation during Laparoscopic Myomectomy: A Retrospective Cohort Study

**DOI:** 10.1155/2017/9068647

**Published:** 2017-12-19

**Authors:** Valerio Mais, Michele Peiretti, Luigi Minerba

**Affiliations:** ^1^Division of Maternal-Fetal Medicine, Department of Surgical Sciences, University of Cagliari Medical School, Monserrato, 09042 Cagliari, Italy; ^2^Department of Medical Sciences and Public Health, University of Cagliari Medical School, Monserrato, 09042 Cagliari, Italy

## Abstract

**Objective:**

To evaluate if improvement of laparoscopic skills can reduce postoperative peritoneal adhesion formation in a clinical setting.

**Study Design:**

We retrospectively evaluated 25 women who underwent laparoscopic myomectomy from January 1993 to June 1994 and 22 women who underwent laparoscopic myomectomy from March 2002 to November 2004. Women had one to four subserous/intramural myomas and received surgery without antiadhesive agents or barriers. Women underwent second-look laparoscopy for assessment of peritoneal adhesion formation 12 to 14 weeks after myomectomy. Adhesions were graded according to the Operative Laparoscopy Study Group scoring system. The main variable to be compared between the two cohorts was the proportion that showed no adhesions at second-look laparoscopy.

**Results:**

Demographic and surgical characteristics were similar between the two cohorts. No complications were observed during surgery. No adverse events were recorded during postoperative course. At second-look laparoscopy, a higher proportion of adhesion-free patients was observed in women who underwent laparoscopic myomectomy from March 2002 to November 2004 (9 out of 22) compared with women who underwent the same surgery from January 1993 to June 1994 (3 out of 25).

**Conclusion:**

The improvement of surgeons' skills obtained after ten years of surgery can reduce postoperative adhesion formation.

## 1. Introduction

Adhesions are the most important complications of intraperitoneal surgery [1, 2]. They often cause hospital readmission due to small-bowel obstruction or chronic abdominal pain [3]. They also increase the operating time and the risk of inadvertent enterotomy in any subsequent surgical procedure due to the attachment of usually separate organs [4–6]. Surgical trauma may lead to ischemia and inflammation and to fibrin persistence in the form of bands that attach opposite peritoneal surfaces [7, 8]. Therefore, late adhesive complications are serious and frequent and should be mentioned during preoperative consent in order to reduce legal implications and litigation [9, 10].

Open surgery seems to be more traumatic; in contrast, laparoscopy has been reported to reduce peritoneal trauma and de novo adhesion formation [2]. However, experimental data suggest that laparoscopy itself may cause peritoneal inflammation due to pneumoperitoneum pressure and duration as well as intraperitoneal CO_2_ concentration, humidity, and temperature [2]. Additionally, the genetic constitutions of individuals undergoing surgery and the laparoscopic skills and training of surgeons have been suggested as cofactors in adhesion formation in laparoscopic rabbit [11–13] and mouse models [14, 15].

To translate discoveries generated in preclinical studies into patient-oriented research, we retrospectively evaluated de novo peritoneal adhesion formation following laparoscopic myomectomy in two cohorts. These cohorts included women who underwent surgery as control groups in two prospective and randomized studies conducted ten years apart, aimed at exploring the efficacy of two different antiadhesion barriers [16, 17]. We aimed to investigate if the improvement of laparoscopic surgical skills obtained by experienced gynecologists after ten years of clinical training in performing the same surgery truly reduced postoperative adhesion formation in women, as already reported in laparoscopic animal models.

## 2. Materials and Methods

### 2.1. Population

We retrospectively compared the postoperative de novo peritoneal adhesion formation assessed in 25 women who underwent laparoscopic myomectomy from January 1993 to June 1994 (cohort 1) with the postoperative adhesion formation assessed in 22 women who underwent the same laparoscopic surgery ten years later (from March 2002 to November 2004) (cohort 2).

These two cohorts of women were selected from our database because they were enrolled as control subjects in two prospective and randomized trials we conducted ten years apart to explore the efficacy of two different antiadhesion barriers [16, 17]. The two trials were conceived and designed by the same investigator (V. Mais), and, therefore, the inclusion criteria and assessment of postoperative adhesion formation were almost identical in both studies [16, 17].

All women had both tubes and ovaries and one to four subserous and/or intramural myomas. The size of the largest myoma ranged from 20 to 60 mm. All women received surgery alone without chemical antiadhesive agents or antiadhesion barriers. Characteristics of CO_2_ insufflations during pneumoperitoneum were the same in both cohorts of women.

Both in 1992 and 2002, the two prospective and randomized studies had the approval of the relevant ethical committees before starting enrollment, and all women were enrolled after written informed consent was obtained.

### 2.2. Surgery

From January 1993 to June 1994, the same surgeon performed laparoscopic myomectomy in all 25 women of cohort 1. Uterine incisions were closed by interrupted sutures with extracorporeal knot tying. Myomas were removed from the peritoneal cavity after morcellation using a manual technique as suggested by Mettler and Semm in 1992 [18].

From March 2002 to November 2004 four different surgeons performed laparoscopic myomectomy in the 22 women of cohort 2. Uterine incisions were closed by the suture technique preferred by each of the four surgeons but this data was not recorded. Myomas were removed from the peritoneal cavity after morcellation with an electromechanical morcellator, as suggested by Carter and McCarus in 1997 [19]. The level of experience of the four surgeons that operated women of cohort 2 was identical because all of them started performing laparoscopic myomectomy in 1993.

All surgeons used dry CO_2_ at room temperature and at the standard intraperitoneal pressure of 12 mmHg to obtain pneumoperitoneum in both cohorts of patients. Blood loss and the duration of surgery from skin incision to closure were recorded in both cohorts of patients.

### 2.3. Assessment of Postoperative Adhesion Formation

In both cohorts of women, assessment of de novo postoperative peritoneal adhesions was obtained by performing a second-look laparoscopy 12 to 14 weeks after myomectomy. The surgeons performing second-look laparoscopy were never the same as the surgeon who performed laparoscopic myomectomy.

In all women of both cohorts, adhesions were scored at 12 sites according to the Operative Laparoscopy Study Group scoring system published in 1991 [20]. The 12 sites were as follows: uterus, right and left ovaries, right and left tubes, omentum, cul-de-sac, both pelvic side-walls, and right and left large bowel and small bowel. Each site was scored as follows: 0, no adhesions; 1, filmy and avascular adhesions; 2, dense and/or vascular adhesions; 3, cohesive adhesions. Scores from all sites were averaged to obtain a total score for each woman.

### 2.4. Statistical Analysis

This study was a retrospective clinical cohort study and so the sample size could not be specified before collecting the data.

The study was informative. All variables were summarized by mean and standard deviation if quantitative, frequencies and percentages if qualitative.

Blood loss and the duration of surgery were compared between the two cohorts of women using *t*-test.

The main variable to be compared between the two cohorts of women who had been identified was the proportion that showed no adhesions (score 0 or adhesion-free women) at second-look laparoscopy. This variable was shown with the confidence interval for proportions according to the method recommended by Wilson [21] with *α* = 0.05.

Luigi Minerba, M.D., Associate Professor of Biomedical Statistics at University of Cagliari Medical School, Italy, reviewed the statistical analysis.

## 3. Results

Demographic and myoma characteristics were similar between the two cohorts of women. The 25 women of cohort 1 had a mean age of 33.2 ± 5.5 years (mean ± SD). The 22 women of cohort 2 had a mean age of 34.0 ± 5.0 years. The mean number of myomas was 2.0 ± 0.9 (mean ± SD) in the women of cohort 1 and 1.7 ± 0.9 in the women of cohort 2. The mean size of the largest myoma was 45.0 ± 9.0 mm (mean ± SD) in the women of cohort 1 and 45.0 ± 10.6 mm in the women of cohort 2.

As for surgical characteristics, the total number and the sites of uterine incisions were similar between the two cohorts of women (Table 1). However, none of the 25 women of cohort 1 had associated lesions requiring concomitant surgery, whereas eleven women of cohort 2 had associated lesions and underwent concomitant surgery, either laparoscopic or hysteroscopic (Table 1). None of the 25 women of cohort 1 and two of the 22 women of cohort 2 had uterine adhesions before myomectomy (Table 1).

Blood loss was similar between the two cohorts of women. The duration of surgery was significantly shorter (*p* = 0.03) for cohort 2 than for cohort 1 (Table 1).

No complications were observed during laparoscopic myomectomy, and no adverse events were recorded during postoperative course in either cohort.

In regard to second-look laparoscopy, the total adhesion scores (mean ± SD) were 1.6 ± 1.0 in women of cohort 1 and 2.1 ± 2.2 in women of cohort 2. The medians of the total adhesion scores were 2 in both cohorts.

**Figure 1:**
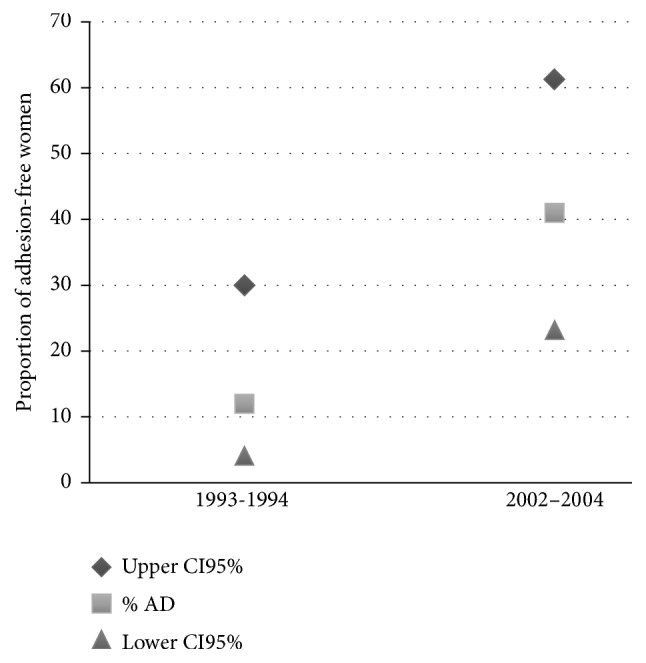
Proportion of adhesion-free women (% AD) in the two cohorts and relative CI_95%_.

A higher proportion of adhesion-free women (score 0, no adhesions) were reported in cohort 2, that is, those who underwent laparoscopic myomectomy from March 2002 to November 2004 (9 out of 22 women, 41%, CI_95_% = 23.2% to 61.3%), compared with cohort 1, who underwent laparoscopic myomectomy from January 1993 to June 1994 (3 out of 25 women, 12%, CI_95_% = 4.1% to 30%) (Figure 1).

## 4. Discussion

To our knowledge this is the first study aimed at translating discoveries generated in preclinical laparoscopic studies using animal models in a clinical setting by evaluating the impact of surgical training on the reduction of postoperative adhesion formation. Indeed, this retrospective cohort study could be included in the first area, or first stage (T1), of translational research according to the NIH definition [22].

Studies in rabbit and mouse laparoscopic models have shown that postoperative adhesions decreased when the number of consecutive laparoscopies performed by surgeons during training increased [12, 13, 15]. Similarly, the data analyzed in our retrospective cohort study suggest that a progressive learning curve of a given laparoscopic surgery, realized in the course of a decade, can result in a noticeable reduction of the formation of postoperative de novo adhesions. Therefore, it is possible for surgeons to learn how to reduce postoperative peritoneal adhesions just as they master the technique of laparoscopic surgery in a clinical setting.

A unique opportunity to compare the results in clinical settings to those suggested by animal model studies was offered by the fact that our research group has conducted two prospective experimental randomized controlled trials on the prevention of de novo peritoneal adhesions obtained with two different adhesion barriers at a distance of 10 years from each other using a virtually identical protocol [16, 17]. In both randomized and controlled prospective studies, we used the same laparoscopic gynecologic surgical model that can cause de novo adhesions. In both studies, postoperative adhesions were evaluated with a second-look laparoscopy performed after the same length of time from the myomectomy intervention and using the same adhesion scoring system published by the Operative Laparoscopy Study Group in 1991 [20]. In both prospective studies, the control group only completed the planned surgery and did not receive any drug or substance that could reduce the formation of adhesions de novo.

The only differences between the two studies that may have had an influence on the formation of postsurgical adhesions were the use of a mechanical morcellator in the first study and an electric morcellator in the second and the concomitance of laparoscopic myomectomy with other laparoscopic or hysteroscopic surgery in about half of the women included in the second study. However, to our knowledge, no study has analyzed the existence of a relationship between the type of morcellator used during laparoscopic myomectomy and incidence of de novo adhesions; postoperative adhesions have been reported to increase when other surgical procedures were associated with laparoscopic myomectomy [23]. Therefore, it might have been expected that the women of cohort 2 would have a higher incidence of postoperative de novo adhesions than women of cohort 1 since no concomitant intervention was made in any patients in cohort 1. Instead, in cohort 2, the percentage of postoperative adhesions was found to be four times less than that in cohort 1.

What distinguishes the two prospective studies and the two cohorts of women included in this retrospective cohort study the most is the elapsed time between the first and the second prospective study. Ten years represents a more than sufficient time to ensure that the gynecologists could better master the technique of laparoscopic myomectomy and consequently become better at reducing the formation of postoperative adhesions. In 1992, laparoscopic myomectomy was still considered an experimental procedure and was performed only in few specialized centers [24]. However, from 2002 to 2004, many gynecologists had already published details of their first 100 or more cases of laparoscopic myomectomy with suggestions regarding intervention optimization [25]. Accordingly, the duration of surgery was significantly shorter for cohort 2 (1993 to 1994) than for cohort 1 (2002 to 2004).

In 2003, the type of uterine suture used during laparoscopic myomectomy was demonstrated to influence the postsurgical adhesion formation [26]. This observation has been confirmed in 2012 by a report on the association between wound appearance after laparoscopic myomectomy and formation of postoperative adhesions even when adhesion barriers were used [27]. Additionally, the number of knots on the uterine suture has been found to influence de novo adhesion formation as a surgical covariate [28].

## 5. Conclusions

A higher proportion of adhesion-free patients were observed in women undergoing laparoscopic myomectomy from 2002 to 2004 than in women operated from 1993 to 1994.

To our knowledge, no study has analyzed the existence of a relationship between surgeon training and peritoneal adhesion formation after laparoscopic surgery in clinical settings. Therefore, our clinical model of de novo adhesion formation, or laparoscopic myomectomy, seems to be the only one to date in which it was possible to show a clear reduction of postoperative adhesions after ten years of technical optimization. However, the results obtained in this retrospective cohort study can be generalized to other types of laparoscopic surgery and may have important implications both in the conceptual interpretation of epidemiological studies on adhesions published in the early 2000s and in the design of new studies on the effectiveness of chemical antiadhesive agents or antiadhesion barriers.

As regards the weight that should be attributed to the results of retrospective epidemiological studies focused on adhesion-related hospital readmissions following abdominal and pelvic surgery published in the early 2000s, it must be emphasized that those retrospective studies included cohorts of patients who underwent initial surgery in the early 1990s. For example, in 2004, Lower and coworkers published the results of an epidemiological study comparing adhesion-related hospital readmissions following laparoscopy or laparotomy and concluded that the rates of adhesion-related hospital readmissions following laparotomy or laparoscopy were similar [29]. However, the two patient cohorts underwent initial open or laparoscopic surgery between April 1996 and March 1997 [29]. It is likely that at the time of initial surgery (1996 to 1997), many surgeons had not yet completed their learning curve for laparoscopic procedures, and, therefore, it is conceivable that a difference between readmission rates following laparotomic surgery and readmission rates following laparoscopic surgery would be observed if the same study were repeated on patient cohorts operated on 10 years later. The confirmation of this hypothesis can be found in a recent meta-analysis that compared the results of laparoscopic treatment of rectal cancer with those of laparotomy. By analyzing randomized clinical trials conducted in the 2000s the authors were able to demonstrate that laparoscopic rectal resection was followed by a smaller percentage of readmissions for bowel obstruction due to adhesions [30].

As for the impact that our retrospective cohort study will have on the design of new prospective studies aimed at evaluating the effectiveness of new strategies for the prevention of postoperative adhesions, we must keep in mind that in the control patients, because they undergo only surgery, the incidence of postoperative adhesions may be reduced after surgeons have completed their learning curve. Therefore, it will be necessary that researchers consider only using clinical models of surgeries definitively validated and sample sizes large enough to be able to identify significant differences in the incidence of adhesions that are not as high as those reported in the past.

## Figures and Tables

**Table 1:** Surgical characteristics of women enrolled in the two cohorts.

Surgical characteristics	Cohort 1 (1993 to 1994) (*n* = 25)	Cohort 2 (2002 to 2004) (*n* = 22)
Total number of uterine incisions^a^	1.4 ± 0.5	1.8 ± 0.9
Anterior incisions^a^	0.4 ± 0.5	0.5 ± 0.6
Fundic incisions^a^	0.5 ± 0.5	0.7 ± 0.6
Posterior incisions^a^	0.4 ± 0.5	0.5 ± 0.8
Women having concomitant surgery	0	11
Women having uterine adhesions before myomectomy	0	2
Blood loss (ml)^a^	197 ± 49	195 ± 57
Duration of surgery (min)^a^	94 ± 29	72 ± 38^b^

^a^Values are means ± SD; ^b^*p* = 0.03.
